# Construction of an evapotranspiration model and analysis of spatiotemporal variation in Xilin River Basin, China

**DOI:** 10.1371/journal.pone.0256981

**Published:** 2021-09-10

**Authors:** Hongbo Yu, Congming Cao, Qiaofeng Zhang, Yuhai Bao

**Affiliations:** 1 School of Geography Science, Inner Mongolia Normal University, Hohhot, China; 2 Inner Mongolia Key Laboratory of Remote Sensing and Geographic Information Systems, Inner Mongolia Normal University, Hohhot, China; Universiti Sains Malaysia, MALAYSIA

## Abstract

Surface evapotranspiration is a water exchange process between the atmosphere, biosphere, and hydrosphere. Accurate evapotranspiration estimations in arid and semi-arid regions are important for monitoring droughts and protecting the ecological environment. The main objective of this study is to build an evapotranspiration estimation model suitable for an effective scientific and objective evaluation of water consumption in the arid and semi-arid regions of the Xilin River Basin based on comprehensive parameters, including meteorological parameters, vegetation coverage, and soil water content. In this study, the community evapotranspiration model was initially constructed using field data, which was then expanded for applicability to the Xilin River Basin based on Geographic Information System technology and spatial heterogeneity characteristics of remote sensing data; both models were significant at the 0.05 level. The monthly evapotranspiration values in July during 2000–2017 and those from April to September (growing season) during the dry, normal, and wet years were calculated using the model at the basin scale. The evapotranspiration showed a generally increasing trend, which was consistent with the fluctuation trend in precipitation in July during 2000–2017. The trend curve for evapotranspiration was gentle during the growing season in dry years, but steep during wet years. The evapotranspiration was the lowest in April, with negligible spatial variations throughout the Xilin River Basin. During May–July, the evapotranspiration was higher than that in other months, in the following order: upper reaches > middle reaches > lower reaches; this was consistent with the vegetation coverage. The evapotranspiration declined and spatial variations were not evident during August–September. The results of this study provide a reference for evapotranspiration model construction and a scientific basis for evaluating regional water resources and protecting the ecological environment.

## 1. Introduction

Surface evapotranspiration (ET), which is the sum of the soil evaporation and plant transpiration, transports moisture from the ground surface to the atmosphere and alleviates net radiation from the surface. Water vapor, accounting for 60–70% of surface precipitation, returns to the atmosphere through ET [1, 2]. ET is typically higher than precipitation in the inland river basins of arid regions [3]. Research on ET has theoretical and practical significance for the elucidation of the mechanisms by which climate change affects the water cycle, the monitoring of agricultural conditions, and the utilization and management of water resources. Relatively rapid and accurate estimations of ET are an important component of ET research.

In contrast to conventional field ET measurement methods, mathematical ET models are advantageous in terms of their convenience, relatively high speed, and low cost. Typically, ET models include single-source models under dense canopy conditions, such as the Penman–Monteith (P–M) model [4, 5], dual-source models under sparse canopy conditions, such as Shuttleworth–Wallace (S–W) model [6], and multi-source models under multi canopy conditions [7]. These models have solid theoretical foundations. Particularly, the P–M model has been applied extensively owing to its high simulation accuracy. However, the P–M model cannot guarantee accuracy when applied to regions with sparse vegetation [8]. The dual-source S–W model concurrently considers soil evaporation and vegetation transpiration, but requires many parameters [9], whose accurate acquisition is complicated (such as the canopy stomatal resistance) [8]. The multi-source models exhibit a similar limitation [8], thus hindering their universal applicability. Therefore, these models are relatively less practical for calculating the actual ET in arid and semi-arid regions owing to the low vegetation coverage. In recent years, the research on ET models has mainly focused on the following aspects: the application of ET models [10–12], the comparison and evaluation of models [13–21], the improvement and optimization of existing ET models [22–26], and the construction of new ET models [10, 16, 20]. However, there are few studies on model construction, some of which use multiple linear regression [16], and some of which use machine learning algorithms such as random forest to estimate ET [10, 20]. The random forest algorithm cannot clarify the explicit relationship between dependent and independent variables, and it requires researchers to have strong computer skills, which greatly limits the application of the model. In many models, it is rare to consider both environmental factors, especially soil moisture data, and biological factors. Therefore, the status of the soil water content and vegetation coverage should be considered for ET modelling in areas where artificial irrigation is not practiced [27]. ET is actually closely related to the vegetation type, leaf area index, vegetation density, plant growth cycle, and soil water content, among other factors [28–32]; therefore, the construction of a simple and practical ET inversion model that considers these parameters is important.

The Xilin River flows through the Xilingol Grassland National Nature Reserve. Additionally, the natural steppes within the Xilin River Basin (XRB) are typical and representative of the Inner Mongolian Plateau, serving as important green barriers for areas such as Beijing and Tianjin [33–36]. Vegetation in this region plays an important role in ecosystems, such as for wind-breaking and sand-fixing, water and soil conservation, and the maintenance of ecological balance. Therefore, the calculation and evaluation of the allocation of water resources in XRB, especially the characteristics of the ET, are significant for the elucidation of the causes of grassland degradation and the formulation of reasonable protection measures.

In this study, a community ET estimation model for the XRB was initially developed using comprehensive parameters, including meteorological parameters, vegetation coverage, and soil water content, based on field observations; model application was then extended to the basin based on Geographic Information System (GIS) technology and the spatial heterogeneity characteristics from remote sensing (RS) data. The ET in the XRB was obtained using the model, followed by an analysis of the spatiotemporal variations in the ET. The results of this study provide a reference for ET model establishment and a scientific basis for protecting the ecological environment and promoting the sustainable development of water resources.

## 2. Materials and methods

### 2.1. Study area

**Fig 1:**
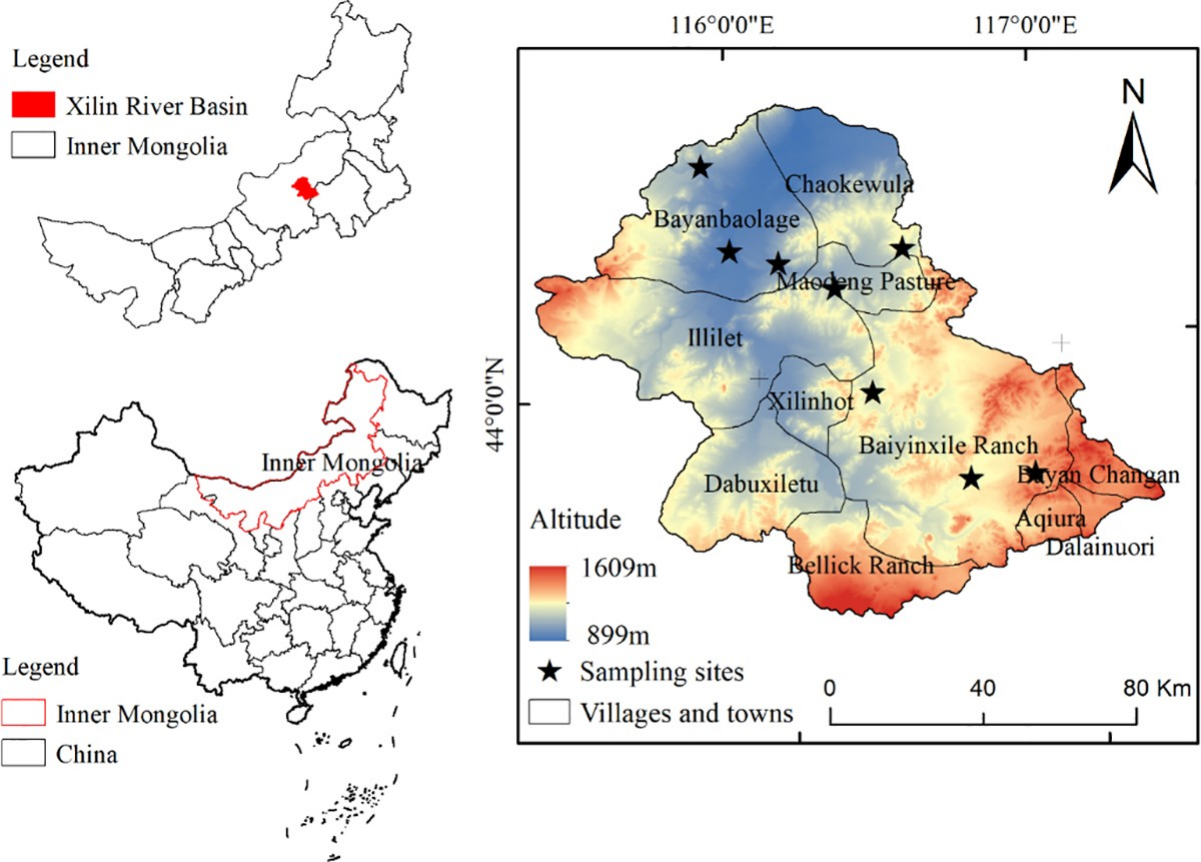
Location, elevation, and field experiment sites distribution map of the Xilin River Basin.

The Xilin River (43°26′–44°39′ N, 115°32′–117°12′ E) originates from the Hexigten Banner in Chifeng City of Inner Mongolia, China. It is an inland river on the Inner Mongolian Plateau whose main trunk spans a total length of 175 km, flowing from the southeast to the northwest at an altitude of approximately 900–1,600 m (Fig 1).

The XRB has a temperate continental climate with warm and humid summers, and cold and dry winters. Significant changes have been observed in the seasonal temperature and precipitation [37]. The soil types, which are distributed sequentially from the southeast to the northwest, include chernozem and dark and light chestnut soils [38]. Chernozem is distributed on the upper reaches of the river at altitudes > 1,350 m, which include the basalt platform in the south and hilly areas in the east. Dark chestnut soil occurs at altitudes of 1,150–1,350 m on the secondary lava platform along the south bank of the river and the vast area along the north bank. Light chestnut and aeolian soils are found in the low hilly areas located in the northwest and middle of the basin, respectively [39].

The XRB predominantly consists of steppes (typical and meadow steppe), which cover more than 85% of the basin [40]. The upper reaches of the XRB comprise *Stipa baicalensis* and *Leymus chinensis* steppe communities; the middle reaches comprise *Leymus chinensis* and *Stipa grandis* steppe communities; and the lower reaches comprise *Stipa krylovii* and *Artemisia frigida* steppe communities [39]. The XRB’s steppe vegetation has gradually degraded in recent years at a degree that intensifies from the upper to the lower reaches. Areas along the river near the Otindag Sandy Land are severely degraded; particularly, the area north of Xilinhot City—the XRB’s northwest region—has become extremely degraded. The middle of the basin is moderately degraded, whereas areas with mild degradation are evenly distributed throughout the basin [41]. Our field survey of the XRB during the vegetation growing season in 2017 showed varying degrees of degradation in most steppe areas.

### 2.2. Data sources

#### 2.2.1. Meteorological data

Meteorological data, including the average temperature, relative atmospheric humidity, actual vapor pressure, actual sunshine duration, and precipitation, were acquired from the China Earth International Exchange Station Climate Data Daily Value Dataset generated by the China Meteorological Data Service Center (http://data.cma.cn/). The daily meteorological data stations and acquisition time are shown in Table 1. The daily meteorological data were used for kriging interpolation.

**Table 1:** Stations and time for meteorological data.

Weather station	From July 1 to July 31, 2000–2011 and 2013–2017	April 1 to September 30, 2000, 2007, 2011 and 2014	April 1–September 30, 2012
Xilinhot City	√	√	√
Abaga	√	√	√
East Ujimqin	√	√	√
West Ujimqin	√	√	√
Hexigten banner	√	√	√
Duolun county	√	√	
Huade county	√	√	
Linxi County			√
Zhenglan banner			√
Zhengxiangbai banner			√

#### 2.2.2. Remote sensing data

The RS images used were obtained from the MODIS satellite data provided by the United States National Aeronautics and Space Administration (NASA) for the following dates: June 25 to August 12 during 2000–2017; March 21 to October 15 for 2000, 2007, 2011, and 2014; and March 29 to October 6 for 2012. The specific products were MOD09A1 and MOD13A1, which combined data from 8 and 16 d, respectively. The satellite orbit was h26v04. After data processing, the data resolution was standardized to 500 m, and the Albers Equal Area Conical projection was employed. The MRT, ENVI5.3, and ArcGIS 10.2 software programs were used for the processing, analysis, and mapping of the MODIS images. The 30 m resolution digital elevation model (DEM) data were obtained from the geospatial data cloud platform (http://www.gscloud.cn/).

Landsat series remote sensing data (column number: 124/29 and 124/30, resolution: 30 m) were selected. ENVI5.3 was used for creating image mosaics, clipping, image enhancement, atmospheric correction, and other preprocessing. According to the first level classification of China’s land resources classification system, the interpretation marks were established based on field investigation, and the remote sensing image maps of the study area on July 18, 2000, August 17, 2005, August 31, 2010 and July 12, 2015 were interpreted using ArcGIS 10.2. Four land use status maps were obtained, and the kappa coefficient determined to evaluate the accuracy reached more than 90%, indicating reliable data. Land use types in the study area include cultivated land, forest land, grassland, water area, residential land, and unused land.

### 2.3. Accuracy evaluation method

The accuracy of the established model of community ET was evaluated using the evaluation indexes of mean square error (MSE), relative error (RE), and consistency index (C-index). The calculation formulae are as follows [42]:

$$MSE=\frac{1}{n}\sum_{i=1}^{n}{\left(x_{i}-y_{i}\right)}^{2}$$ (1)

$$RE=\frac{\left|x_{i}-y_{i}\right|}{x_{i}}\times 100\%$$ (2)

$$C‐index=1-\frac{\sum_{i=1}^{n}{\left(x_{i}-y_{i}\right)}^{2}}{\sum_{i=1}^{n}{\left(\left|x_{i}-\bar{x}|+|y_{i}-\bar{y}\right|\right)}^{2}}$$ (3)

where x_i_ is the measured value of group i, y_i_ is the model estimate of group i, $\bar{x}$ is the average value of x_i_, $\bar{y}$ is the average value of y_i_, and n is the number of samples. The smaller MSE and RE, and the closer C-index is to 1, which indicates the higher accuracy of estimation.

## 3. Model construction

### 3.1. Evapotranspiration model construction for vegetation communities

**Fig 2:**
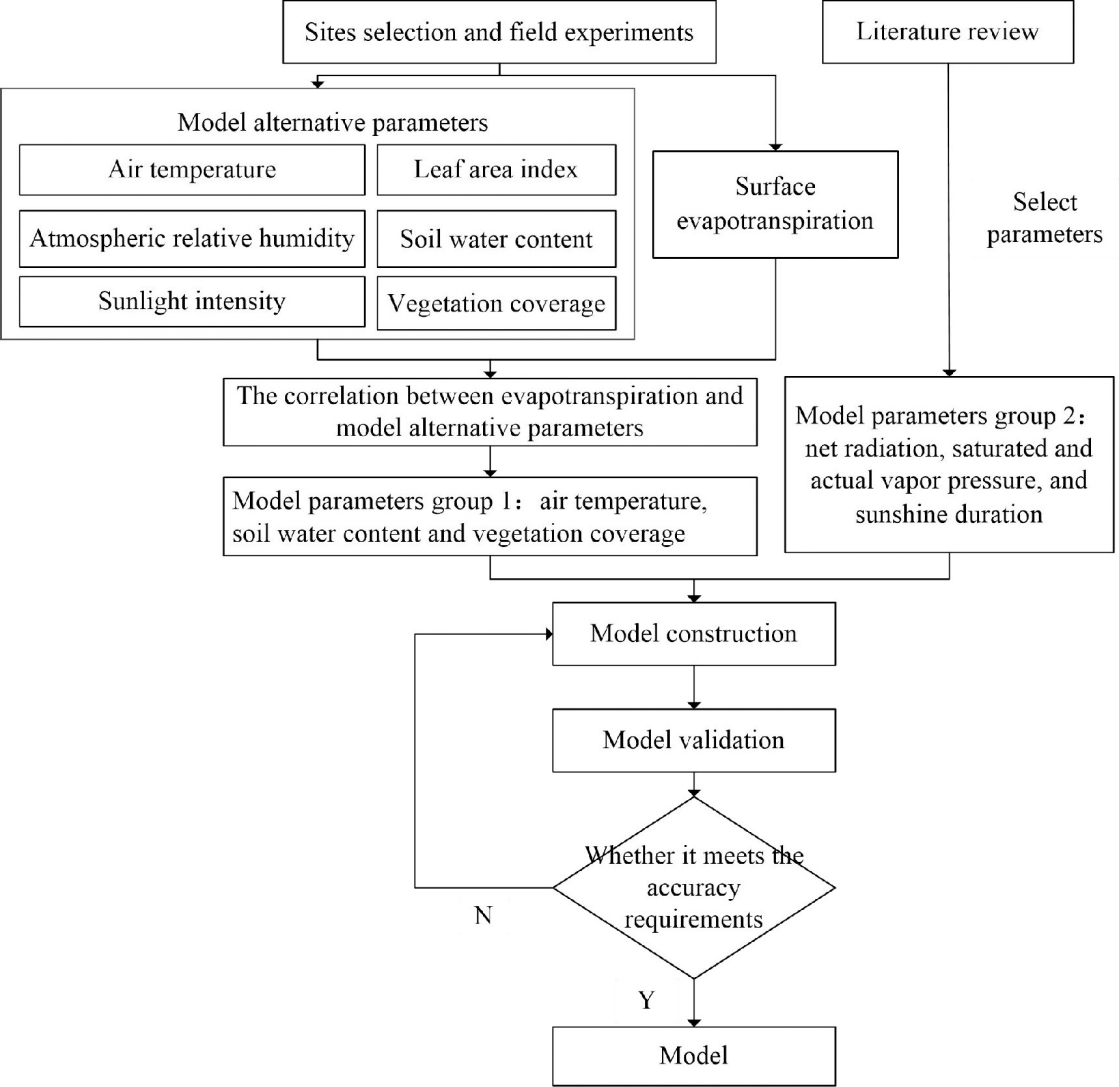
Flow chart of modeling method.

The process of modeling is shown in Fig 2. The field experiments were carried out during the plant growing season from May to September, 2017 (field experiment sites are shown in Fig 1, and the supplementary measurement of data was carried out in 2019). Two experiment sites in the southern part of Baiyinxile Ranch, located in the upper reaches of the XRB, represent mildly and severely degraded meadow steppe communities (the upper reaches are the more degraded steppes), with a chernozem soil type. Three experiment sites were selected from the areas surrounding Xilinhot City and the Maodeng Pasture, all are located in the middle reaches. These sites represent non-degraded and mildly degraded *Leymus chinensis* steppe communities and mildly degraded *Stipa grandis* steppe communities, with a dark chestnut soil type. Three additional experiment sites were selected at Bayanbaolage Sumu in the lower reaches. These are all *Stipa krylovii* steppe communities growing in light chestnut soil, where one site is non-degraded and the other two sites are severely degraded. There were eight experiment sites in total throughout the XRB.

The microlysimeter method (weighing of soil columns) was used to determine the daily ET. At each experiment site, a location with uniform plant growth was selected. An iron bucket with a diameter of 25 cm and depth of 30 cm was filled to form a circular soil column of the same volume, without destroying the soil structure and vegetation. Next, the iron bucket was buried parallel to the ground surface at the observation site to simulate the actual ET. The soil column was weighed daily at 20:00, and the daily ET of the community was determined based on the changes in the weight of the column. The meteorological conditions, soil water content, vegetation coverage, and other data during the same period were measured using a portable temperature–humidity optical data logger (DJL-18, Zhejiang TOP Cloud-agri Technology Co., Ltd.), rapid soil moisture detector (TZS-1K, Zhejiang TOP Cloud-agri Technology Co., Ltd.), and the community survey method. Field experiments were conducted at each site 2–3 d per month. The experiments were repeated thrice at each site to obtain an average value. A total of 102 datasets were obtained: 20 sets were randomly selected for model verification and 82 sets were used to build ET models for the vegetation communities.

The actual ET is closely related to the net radiation, temperature, saturated and actual vapor pressure, and sunshine duration [39, 43–47]. In addition to the meteorological factors, the soil moisture and vegetation characteristics, mainly including the vegetation coverage, were also considered in the model. The goal was to build ET models suitable for the vegetation communities in the study area with few parameters, less complications, and high simulation accuracies, while considering the impact of meteorological, soil, and vegetation factors.

After the observed data were processed to obtain the requisite factors stated above, the screening, model building, and verification steps were carried out in cycles. After repeated verifications and comparisons, the following models were built:

$$Model\;1:\;ET=\frac{R_{n}\times \Delta \times T\times SCD\times w_{s}}{\Delta +\gamma }$$

$$Model\;2:\;ET=\frac{R_{n}\times T\times SCD\times w_{s}}{\Delta +\gamma }$$

$$Model\;3:\;ET=\frac{{(R}_{n}\times \Delta +T)\times SCD\times w_{s}}{\Delta +\gamma }$$

$$Model\;4:\;ET=\frac{{(R}_{n}+\frac{\gamma }{T})\times SCD\times w_{s}}{\Delta +\gamma }$$

$$Model\;5:\;ET=\frac{{(R}_{n}+\frac{\gamma }{T})\times SCD\times w_{s}}{\gamma }$$

Model…:Mixedcalculationsofthevariousfactors

where R_n_ is the net radiation (MJ m^–2^ d^–1^), T is the average temperature during the day (°C), SCD is the vegetation coverage (from 0–1), w_s_ is the volumetric soil water content (from 0–1), Δ is the slope of the saturated vapor pressure and temperature curve (kPa °C^–1^), and γ is the hygrometer constant (kPa °C^–1^). Table 2 presents the results of these models.

**Table 2:** Results of the accuracy analysis and error verification for the various models.

Test	Model 1	Model 2	Model 3	Model 4	Model 5	Model…
Regression coefficient	0.15	0.03	0.34	0.71	0.17	/
Regression intercept	0.39	0.37	0.30	0.34	0.35	/
Coefficient of determination (R^2^)	0.7926	0.7903	0.7796	0.7679	0.7932	< 0.6
Hypothesis test (F value)	305.7	301.6	282.9	264.7	306.8	/
Consistency index (C-index)	0.970	0.954	0.934	0.921	0.948	/
Mean square error (MSE)	0.062	0.092	0.146	0.151	0.105	/
Relative error (RE)	14.03%	17.96%	21.96%	25.35%	19.43%	> 26%

**Fig 3:**
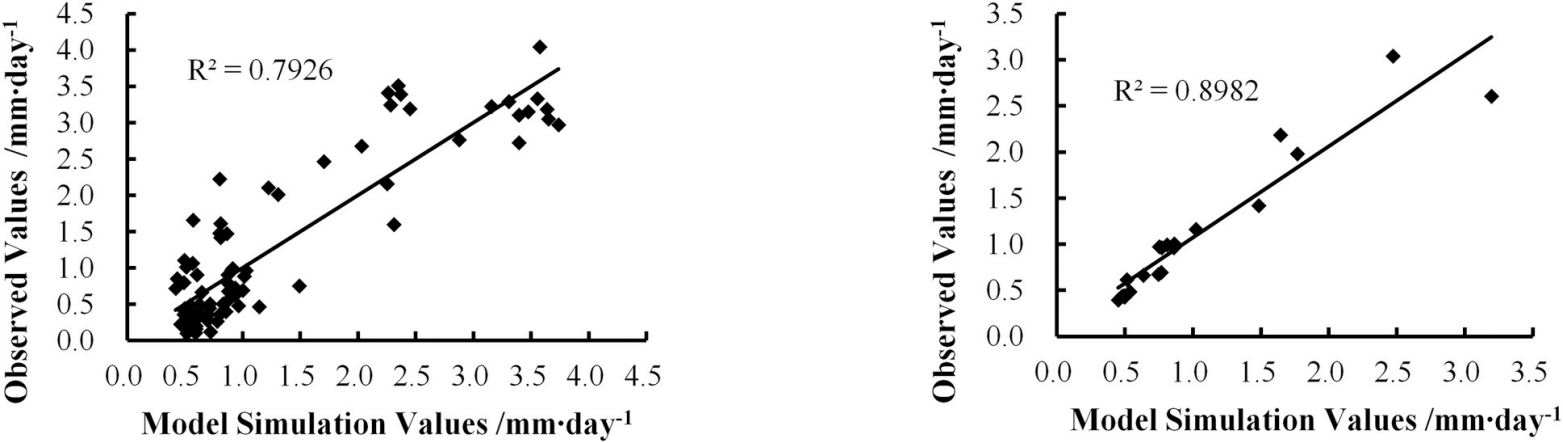
Comparison between the estimated and observed daily evapotranspiration values of the vegetation communities: (a) 82 sets of modeling data and (b) 20 sets of verification data.

Fig 3(A) shows the results of Model 1, constructed with 82 datasets, while Fig 3(B) shows the result of Model 1, as verified by the 20 datasets. A comparison of the models (Table 2 and Fig 3) shows that Model 1 has a relatively high fitting accuracy, with an R^2^ of 0.7926. The F value was 305.7, which was significantly higher than the critical value of 2.63 at the P = 0.05 significance level. The verification result of this model yielded the lowest error, with C-index and R^2^ values of 0.97 and 0.8982, respectively. The MSE was 0.062 and the average RE was 14.03%. These results indicate that the model performance measures satisfy the accuracy requirements for the estimation of the ET from the vegetation communities in the study area. Therefore, Model 1 was selected as the final model for use in this study:

$${ET}_{s}=a\frac{R_{n}\times \Delta \times T\times SCD\times w_{s}}{\Delta +\gamma }+b$$ (4)

where ET_s_ is the model-estimated daily ET (mm d^–1^); and a and b are the correction coefficients for the XRB, where a = 0.15 and b = 0.39. The calculation methods for certain model parameters are described in detail in our previous study [48].

### 3.2. Up-scaling ET estimation model from vegetation community scale to basin scale

The ET mechanism remains unchanged when it is scaled up from the vegetation communities to the entire XRB, mainly reflecting the spatial heterogeneity. The RS and GIS platforms provide robust technological support for such scale expansions. Furthermore, RS images can reflect the characteristics of spatial heterogeneity. In this study, the final ET model for the vegetation communities was applied to the entire XRB. Data on soil water content (Eq 5) and vegetation coverage (Eq 6) were obtained via the inversion of the satellite data; the point data for the meteorological factors were interpolated to obtain the surface data. The ENVI5.3 and ArcGIS 10.2 software were used to process the data at the pixel level to obtain the ET of the XRB.

#### 3.2.1. Soil water content

Bands 6 and 7 of the MOD09A1 product were used for the inversion of the surface water capacity index (SWCI). The SWCI is calculated as follows:

$$SWCI=\frac{b_{6}-b_{7}}{b_{6}+b_{7}}$$ (5)

where b_6_ and b_7_ are the reflectivity values of bands 6 and 7, respectively.

#### 3.2.2. Vegetation coverage

Vegetation coverage refers to the plant–soil ratio, which is the proportion of the total soil area occupied by the vertically projected area of the vegetation canopy, calculated as follows:

$$SCD=\frac{NDVI-{NDVI}_{min}}{{NDVI}_{max}-{NDVI}_{min}}$$ (6)

where NDVI is the normalized vegetation index of the MOD13A1 product, and NDVI_max_ and NDVI_min_ are the NDVI values of areas with full and no vegetation cover, respectively. Based on field investigations and a comparative analysis with the satellite images, in this study, the NDVI_max_ and NDVI_min_ values were calculated as 0.68 and 0.03, respectively. If NDVI > NDVI_max_, then SCD = 1; if NDVI < NDVI_min_, then SCD = 0.

#### 3.2.3. Verification of estimated evapotranspiration results

**Fig 4:**
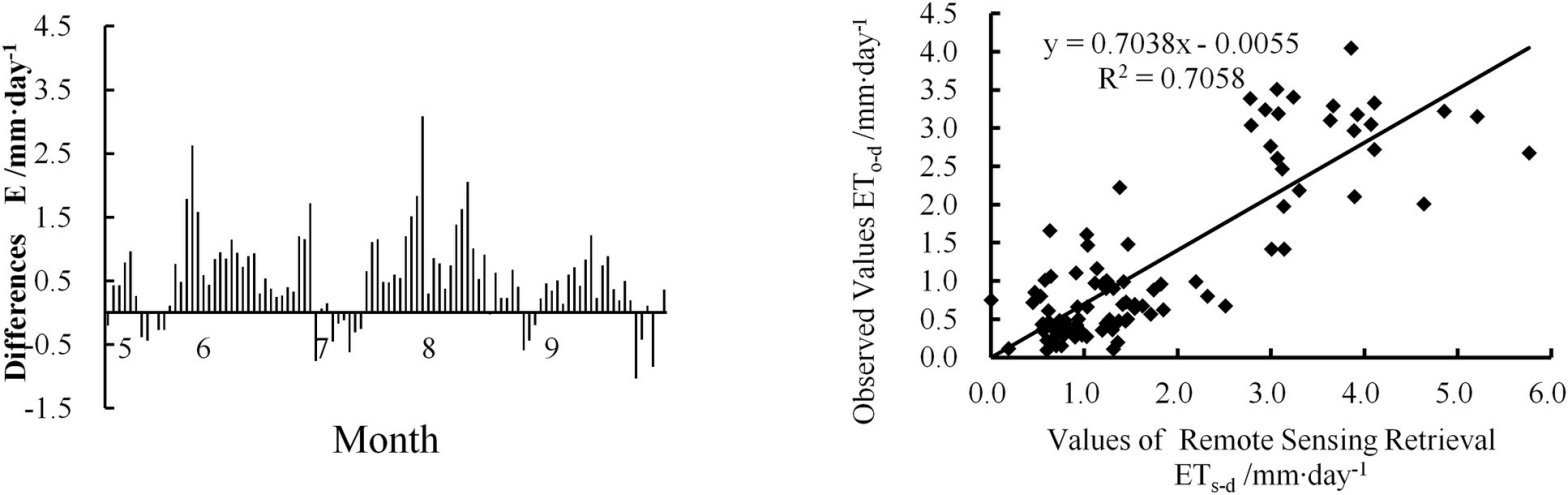
Comparison between the estimated and observed ET: (a) distribution map of the difference between the estimated and observed daily ET and (b) correlation between the estimated and observed daily ET.

The observed daily ET data (ET_o-d_) for May–September 2017 were compared with the daily ET (ET_s-d_) obtained using the up-scaling ET estimation method described above. Fig 4(A) shows the distribution of the difference (E value) between the ET_s-d_ and ET_o-d_ values. The ET_s-d_ value of most points was slightly higher than the ET_o-d_ value, with only 20% of the ET_s-d_ values being slightly lower than the ET_o-d_ value. Numerically, the overestimated values were closer to ET_o-d_ than the underestimated values. The ET_s-d_ value of one-third of the samples from May and July was lower than their ET_o-d_ value. In May, these samples were concentrated on the degraded *Stipa krylovii* steppe areas in the lower reaches. In July, they were concentrated on the *Leymus chinensis* and *Stipa grandis* steppe areas in the middle reaches. Fig 4(B) shows the correlation between the model-estimated ET_s-d_ and observed ET_o-d_, with an R^2^ of 0.7058, indicating that the model-estimated ET is applicable in the study area.

## 4. Results

The monthly data from bands b6 and b7 in the mod09a1 product and the NDVI band of the mod13a1 product were synthesized via the maximum synthesis method. The monthly soil water content and vegetation coverage data were calculated using Eqs (5) and (6). Combined with the monthly meteorological data, the monthly ET (ET_m_) was obtained using the basin-scale ET estimation method.

Based on GIS and RS techniques, pixel-by-pixel calculations were performed using the model before determining the monthly ET values in July during 2000–2017 and the monthly ET from April to September during the growing season of dry (2000 and 2007), normal (2011 and 2014), and wet years (2012).

Using the interpreted land use vector map to extract the grassland range from the basin ET data, the ET data for grasslands were obtained. According to the comparative analysis of remote sensing images and vector images, combined with the principle of time proximity, the ET of grasslands from 2000 to 2003 were extracted using the land use vector map of 2000, those from 2004 to 2007 were extracted using the land use vector map of 2005, and those from 2008 to 2011 were extracted using the land use vector map of 2010. The land use vector map of 2015 was used to extract the ET of grasslands from 2012 to 2017 and the multi-year average ET of grassland for July 2000–2017.

### 4.1. Spatial variations in evapotranspiration

**Fig 5:**
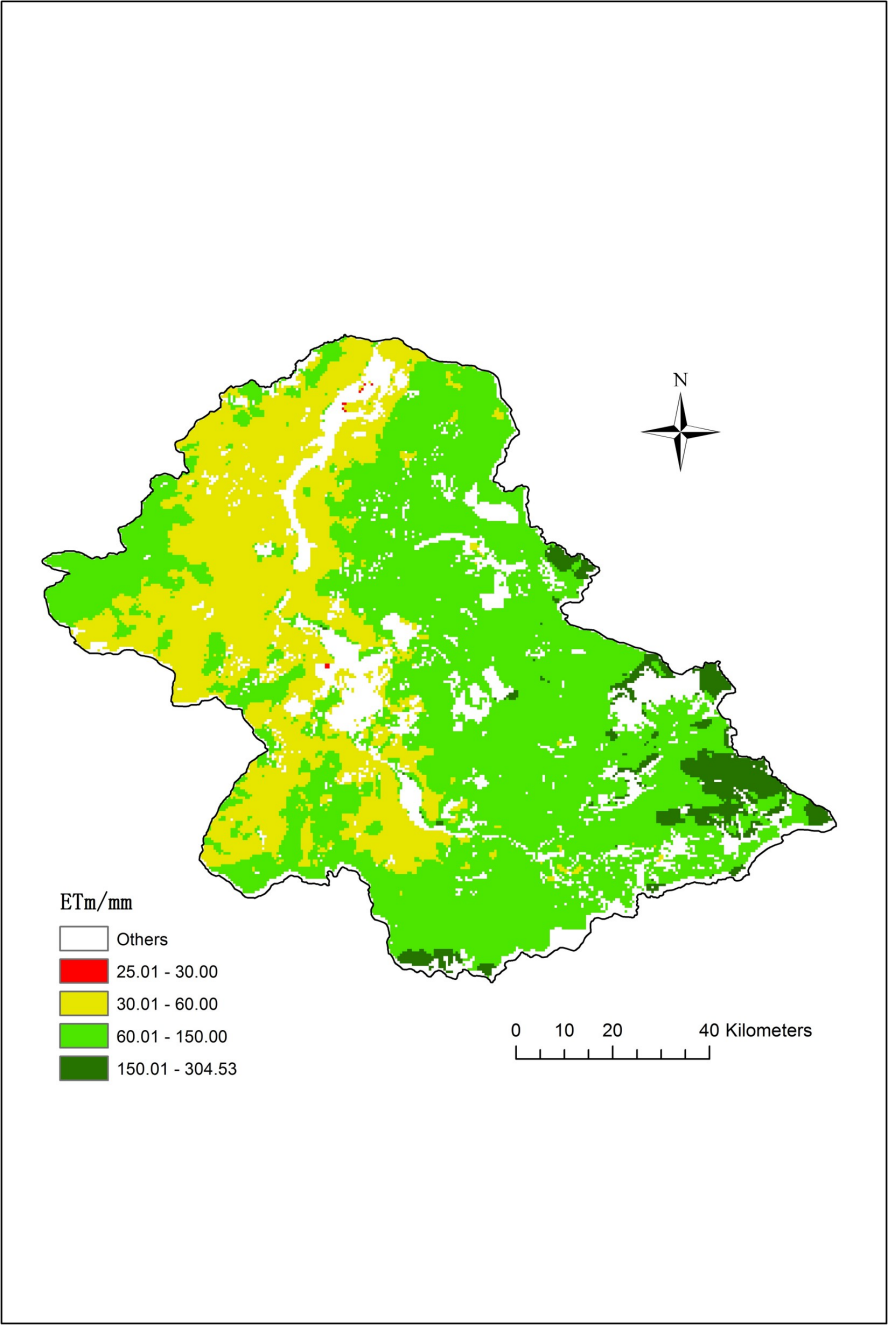
Average monthly ET in July during 2000–2017.

The multi-year average ET_m_ of the XRB for July 2000–2017 exhibited strong spatial heterogeneity (Fig 5). The difference between the maximum and minimum values (304.53 and 25.01 mm, respectively) amounted to 279.52 mm, with an MSE of 34.81 mm. Owing to the different types of steppe and soil, the ET throughout the entire XRB in July showed a ribbon-like distribution that decreased from the southeast to the northwest. The ET was the highest in the meadow steppes (*Stipa baicalensis* and *Filifolium sibiricum*) in the upper reaches. The multi-year average ET for these areas in July was > 150 mm. This was followed by that of the typical steppes in the middle reaches with a multi-year average ET in July of 60–150 mm. ET was the lowest in the *Stipa krylovii* steppe areas in the lower reaches and for land with saline soil [39]. The multi-year average ET in these areas in July was < 60 mm. These results are consistent with the field observations conducted by the project team in 2017: the spatial distribution pattern of ET was upper > middle > lower reaches.

**Fig 6:**
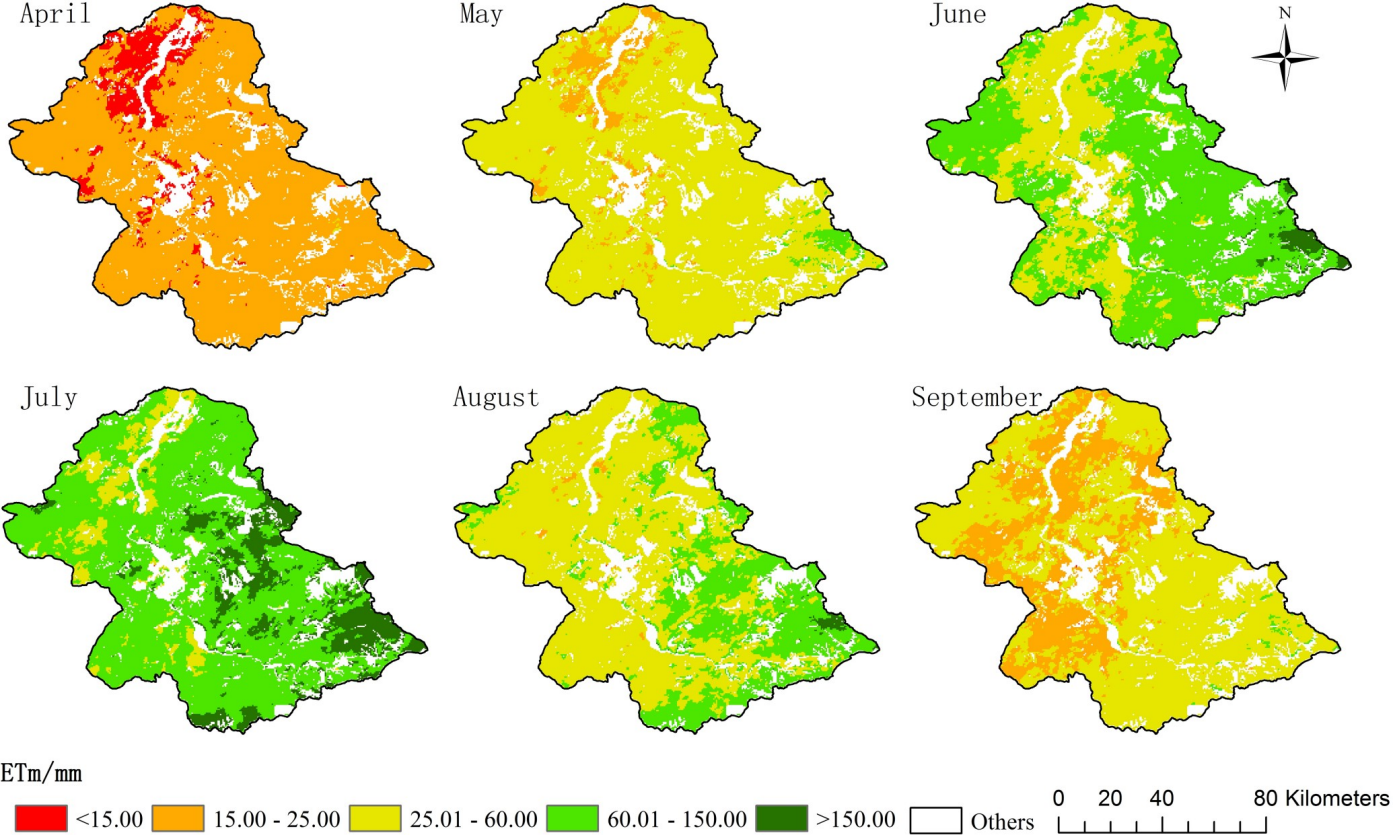
Monthly evapotranspiration in the Xilin River Basin from April to September 2014.

During the plant growing season, the spatial distribution of the ET in the XRB was consistent with the phenological period of vegetation. A normal year, e.g., 2014, was used as an example, and the classification threshold was unified (Fig 6). The ET in April was relatively low, and there was no notable spatial heterogeneity. At this time, the vegetation cover was undergoing the regreening stage [49]. Following rapid vegetation cover growth during May–June, the ET of the meadow steppes in the upper reaches began to increase. The ET in the XRB increased overall, and the spatial pattern of the ET gradually developed. July was the peak season for plant growth in the XRB, when both the transpiration and evaporation were strong, i.e., the ET in most areas exceeded 60 mm. The vegetation entered a period of decline and yellowing during August–September. The ET gradually decreased, with low-value areas gradually spreading from the lower to middle and upper reaches, and then returning to a uniform spatial distribution. The plants began withering in the later part of the growing period, and herders in parts of the eastern region of the XRB tend to cut hay during late summer [50, 51]. Consequently, there was a gradual increase in the proportion of soil evaporation in the total ET.

### 4.2. Temporal variations in evapotranspiration

**Fig 7:**
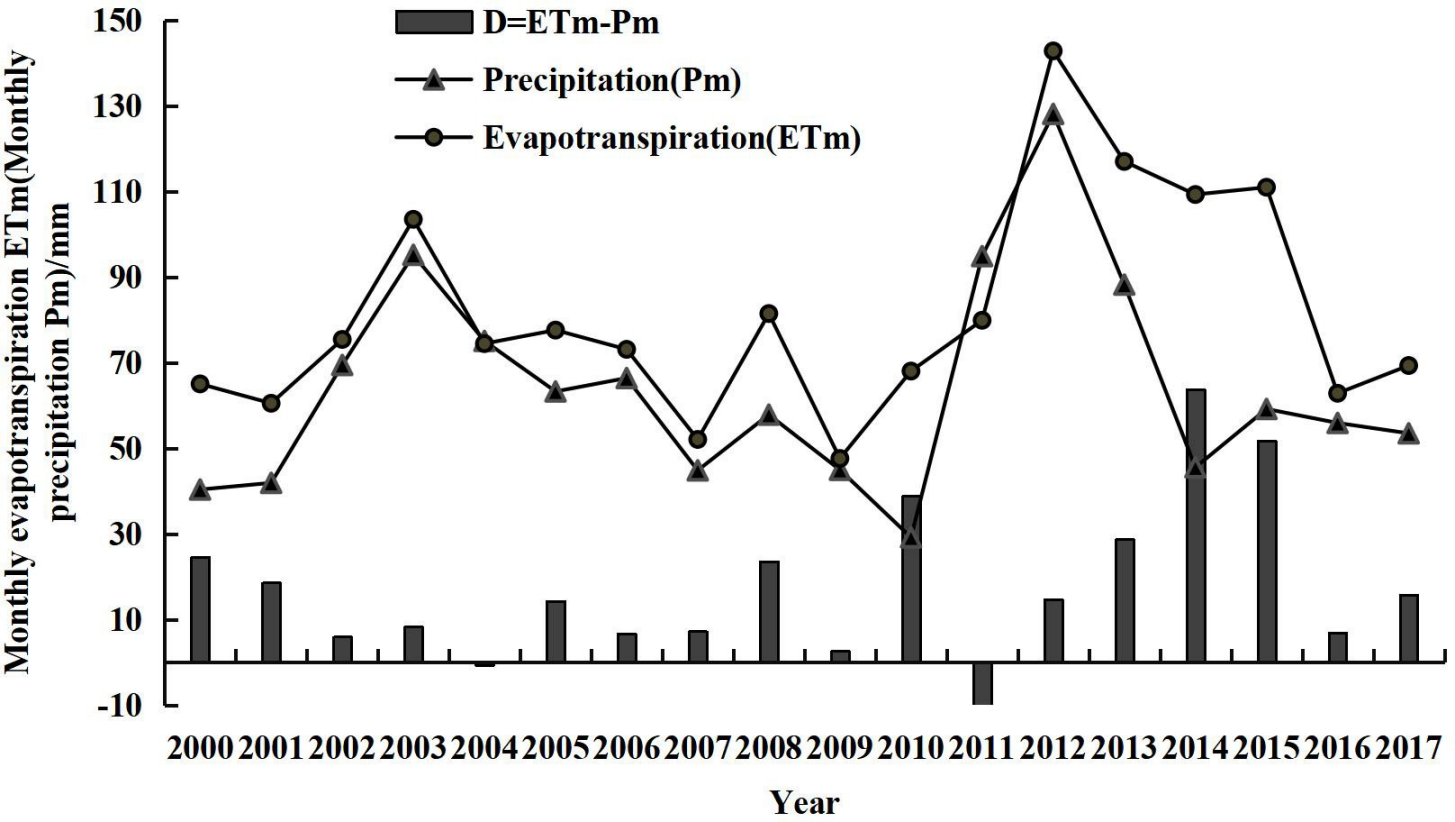
ET and precipitation changes in July during 2000–2017.

Substantial interannual fluctuations were observed in the monthly evapotranspiration (ET_m_) in July from 2000 to 2017 (Fig 7). The minimum value of the curve (47.62 mm) was recorded in 2009, followed by that in 2007, whereas the maximum value (142.83 mm) was recorded in 2012, followed by that in 2013. The multi-year average ET in July was 81.93 mm. Affected by precipitation, the average ET in July (2000–2017) presented an increasing trend over the years, although the trend was not significant (p > 0.05). The difference (D) between the ET and precipitation in July during the study period also showed variations. Fig 7 shows that D in July 2011 was the smallest and negative, whereas that in July 2014 was the largest.

**Fig 8:**
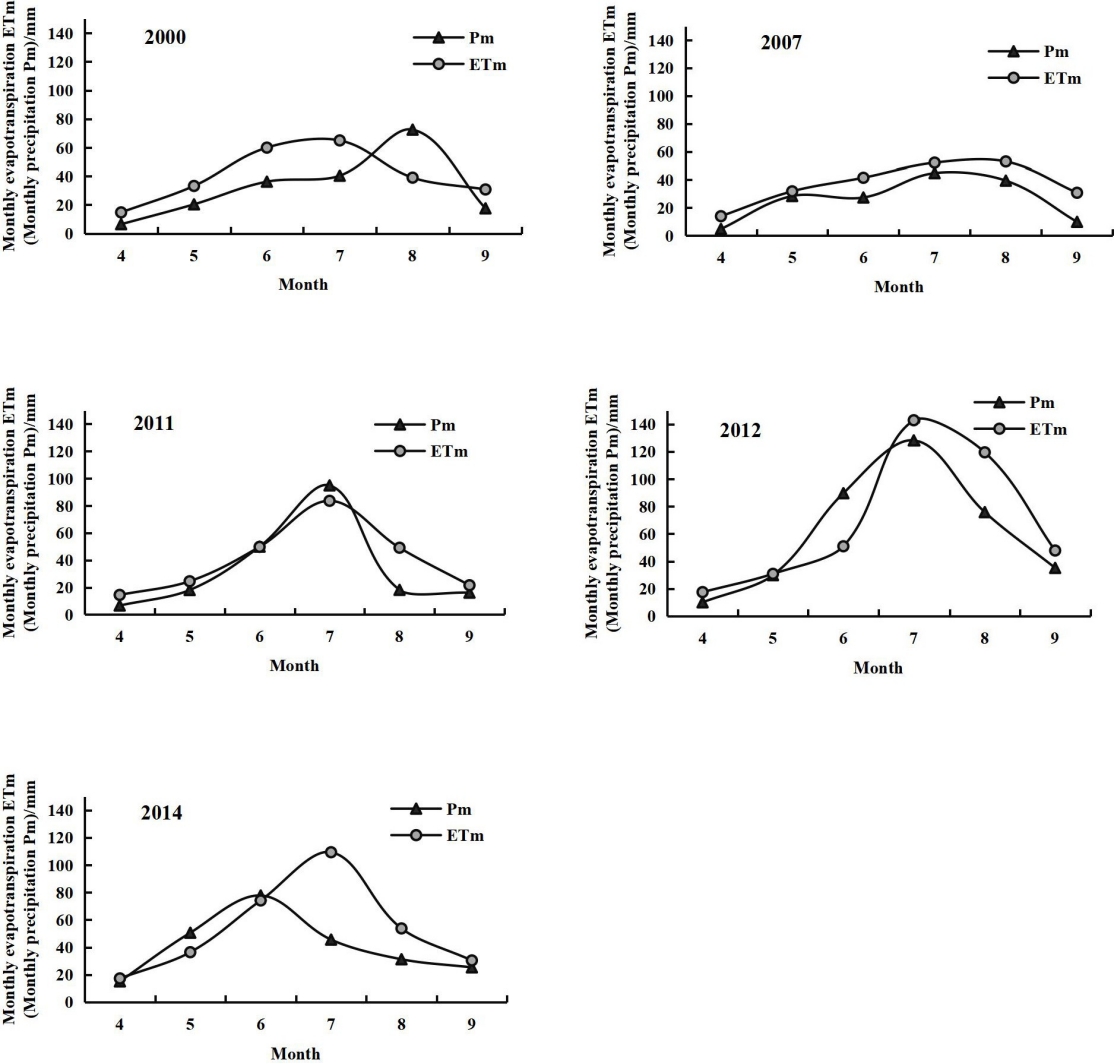
Changes in the evapotranspiration and precipitation during the growing seasons of different years.

The ET values in the growing season of the years with different precipitation types also showed variations. Considering the dry and wet years of 2007 and 2012, respectively, as examples, the ET curves of both years first increased before decreasing, forming a single peak (Fig 8). The variations in the curve were gradual in 2007, the peak was not notable in August, and the trend was similar to that of the precipitation curve. The monthly ET was always greater than the monthly precipitation, indicating that precipitation could not fully satisfy the demand for plant transpiration and soil evaporation during dry years. Vegetation was almost constantly under water stress throughout the growing season. In the natural steppes without artificial irrigation, the ET mostly originated from the soil water content [43]. ET variations during the growing season in 2012 exhibited a distinct bell-shaped curve. The ET increased slowly from April to June, increased sharply in July to reach the peak, and then rapidly declined during August–September. The variations in the ET curve were consistent with the distribution of precipitation and the plant growth cycle. The variations during the growing season in normal years ranged between the values of the dry and wet years.

Precipitation in July 2011 was greater than that in July 2014, but the ET was lower. Fig 8 shows that maximum precipitation in 2011 was recorded in July, whereas that in 2014 was recorded in June. Furthermore, the cumulative precipitation amount during April–June 2014 (143.35 mm) was higher than that during April–June 2011 (74.56 mm). If the precipitation distribution is uniform during the rapid growing season, plants can obtain an adequate amount of water. The average coverage throughout the XRB in July 2011 and 2014 was 0.31 and 0.35, respectively. Plant growth was thus more rapid and the leaves were broader in July 2014. Following increased transpiration, the ET naturally increased rapidly [52]. Hence, the amount of ET is simultaneously affected by the plant characteristics and moisture. The ET was closely related to precipitation in the same period, and affected by the cumulative precipitation in the previous period.

Table 3 presents the total ET (ET_t_) for grasslands and precipitation (P_t_) in the XRB from April to September for different years. For both the P_t_ and ET_t_ during the growing season, the ranking of the years was 2012 > 2014 > 2011 > 2000 > 2007. This indicates that precipitation significantly affected the ET. In arid and semi-arid areas, the ET conformed to the characteristics of wet > normal > dry years.

**Table 3:** Precipitation and total evapotranspiration during the growing season.

Year	2000	2007	2011	2012	2014
**Total ET (ET**_**t**_ **/mm)**	242.86	223.00	242.91	409.23	321.08
**Precipitation (P**_**t**_ **/mm)**	193.62	154.69	203.69	368.44	245.41

## 5. Discussion

Further analysis of the ET data retrieved from the model and environmental factors in the same period showed that monthly ET in the XRB was significantly correlated with soil water content, vegetation coverage, precipitation, and air temperature, with correlation coefficients of 0.40, 0.78, 0.63, and 0.48, respectively. They were all significantly correlated (P < 0.05). Wang et al. [53] found that the ET of *Leymus chinensis* steppes was significantly correlated with the soil water content (R^2^ = 0.85), such that they established a linear regression equation for the daily ET with the soil water content and average temperature. Zhang [54] studied the response of the ET from *Haloxylon ammodendron* plantations to environmental factors, concluding that the soil water content was the main environmental factor affecting the variations in the daily ET. In comparison, the relative humidity and wind speed had the lowest impacts on the ET.

The main heat factors considered in the model are temperature and net radiation, which are closely related to the actual evapotranspiration. As the driving force of ecosystem operation, net radiation can change the distribution of water and heat fluxes and the change in water phases, thereby affecting changes in ET [55, 56]. Differences in vegetation coverage may lead to differences in the absorption of net radiation by the surface. Higher vegetation coverage is conducive to the absorption of net radiation energy received by the surface, thereby increasing transpiration. Therefore, the information on temperature, net radiation and vegetation coverage was added into the model. The ET was most closely related to moisture, heat, and vegetation growth. Different factors can change the water and heat status of soil and plants in different manners, subsequently affecting soil evaporation and plant transpiration. Thus, in addition to meteorological factors, the soil and vegetation factors should be considered during modeling. In this study, point and surface data, observation data, and RS and GIS technologies were combined to explore the feasibility of estimating ET. The ET estimation method can objectively reflect the moisture dissipation and its spatiotemporal distribution in the area.

Liu et al. [47] used an eddy correlator and a large-scale weighing lysimeter to determine the daily ET, finding that the correlation between the daily ET and average daily wind speed ranked last among the correlations between all meteorological factors; the relationship between the two was not significant. Yu et al. [57] discovered that the effect of the wind speed on transpiration is complex; although wind speeds within a specific range are positively correlated with the sap flow of trees, excessive wind speeds reduce or even close the stomatal opening. Therefore, the wind speed was not measured during the field experiments or considered during model development in this study.

The proposed model was then used to analyze the characteristics of the spatiotemporal variations in the ET throughout the XRB during 2000–2017. The results indicated that the monthly ET was greater than the precipitation during the same period. Moisture dissipation was greater than replenishment during dry years, even during years when there was a water deficit throughout the area. Although the XRB is in an arid and semi-arid climatic zone, with precipitations as the main source of moisture replenishment, the soil water content can also be used as a moisture source for ET. The main source for the soil water content is precipitation during the late autumn and winter of the previous year, as well as accumulated precipitation from earlier years [58]. This is consistent with the findings of previous studies [59–61].

Although this study revealed significant findings, a considerable amount of research remains to be conducted. The ET estimation model developed in this study using observation data is an empirical model. Although the model was verified based on comparisons and only yielded small errors, it continues to be limited in the theoretical sense compared to the mechanism model, especially in terms of clarifying the principle and process of ET. Therefore, the ET model needs to be modified for its application to other arid and semi-arid areas. Due to different climate conditions, there are some differences in the underlying surface environment such as soil and vegetation, but the dominant factors that affect ET are basically the same [56], including temperature, water conditions, solar radiation, and vegetation factors. Therefore, the main part of the model remains unchanged, but the a and b coefficients need to be amended.

The heat, moisture, vegetation, and other model parameters were calculated by a variety of linear and nonlinear combination methods. After comparing and analyzing the simulation errors of various models, the model with the highest accuracy was selected. However, the modeling was not comprehensive, for example, there was no single soil evaporation test on bare land, so there is an uncertainty in the application of the model in areas without vegetation coverage. The study area is in the arid and semi-arid grassland area in northern China. The growing season of plants is from April to September every yea. The model is not suitable for application in the non-growing season of vegetation. Therefore, the application of the model has some limitations in time and space, which need to be improved in the future.

Future research may increase the spatial coverage of the observation sites, allowing for improved simulations of the ET in the XRB. The number of years and duration of the experimental period can also be increased, e.g., continuous observations of the meteorological conditions and soil water content can be performed. New measurement equipment and methods can be introduced to improve the simulation accuracy and precision. In future research, additional data, such as the degree of steppe degradation and the grazing intensity and duration, should be included to further analyze the relationship between human activities and surface ET. Subsequently, the patterns may be summarized to allow the findings to guide the management of regional water resources and studies on the causes of droughts.

## 6. Conclusions

In this study, the XRB was used as the study area. Based on GIS technology and the spatial heterogeneity characteristics of RS data, an ET estimation method suitable for this region was proposed with reference to comprehensive parameters, including meteorological parameters, vegetation coverage, and soil water content. The ET in the XRB was obtained via pixel-by-pixel calculations, followed by an analysis of the spatiotemporal variations in the ET. The model exhibited a high fitting accuracy at the vegetation community scale, with an R^2^ value of 0.7926. The model was extended from the community to the entire basin, with a verification accuracy of R^2^ = 0.7058. Both models were significant at the 0.05 level and applicable for the estimation of ET in the XRB.

As the ET is closely related to moisture, heat, and vegetation growth, the ET model that considered the meteorological, soil, and vegetation characteristics, yielded relatively more accurate, effective, scientific, and objective ET measurements. This model required a few easily available parameters, indicating its potential for practical applications.

This model can be used as a reference for the establishment of ET models in other areas with similar climates, sparse distribution of meteorological stations, and low vegetation coverage. The future planning of ET models in this kind of environment should aim to improve the estimation accuracy of soil evaporation through quantitative experiments. By understanding the surface evapotranspiration water consumption, we hope to help improve the water resource management in these areas and to ensure the sustainable utilization of water resources.
